# Elbow dislocation with lateral condyle and coronoid fractures

**DOI:** 10.1080/23320885.2022.2109473

**Published:** 2022-08-12

**Authors:** Yousef Fallah, Behnam Baghianimoghadam, Seyed-Aref Daneshi

**Affiliations:** a Department of Orthopedic Trauma Surgery, Sina Hospital, Tehran University of Medical Sciences, Tehran, Iran; b Department of Orthopedic and Trauma Surgery, Tehran University of Medical Sciences, Tehran, Iran

**Keywords:** Elbow dislocation, lateral condyle fracture, coronoid fractures, fixation

## Abstract

Elbow dislocations are simple or complex types. We introduce a patient with the elbow dislocation, lateral condyle fracture and coronoid fracture and their surgical management. This type of fracture is rare in adults. Particular attention should pay to coronoid fracture repair during the treatment.

## Introduction

Elbow instability has been the subject of many challenges, and there is still much controversy over its diagnosis, evaluation and treatment [1]. Terminologically, simple elbow dislocations do not involve bone damage associated with the dislocation, and parts of the stabilizing soft tissue around the elbow are damaged. While in complex dislocations, there is bone damage and also soft tissue damage.

Many elbow dislocations are associated with minor bone injuries due to incisional injuries or fractures. Some of these fractures can weaken the prognosis of treatment, while the presence of others has little effect on the type of treatment or prognosis. Here, we introduce a patient with the elbow dislocation, lateral condyle fracture, coronoid fracture and surgical management of this complex injury.

## Case presentation

**Figure 1.**
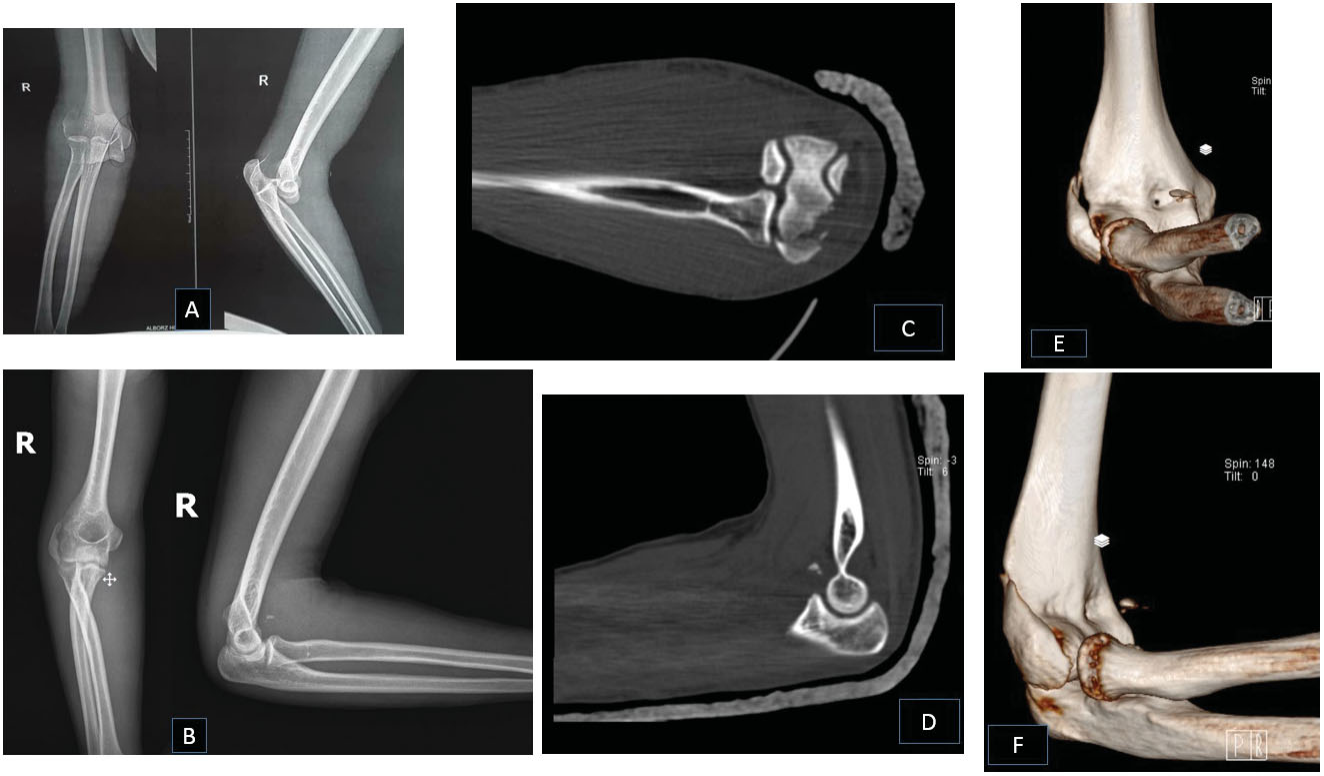
The primary radiographs of the patient before (A) and after (B) close reduction. Axial (C), sagittal (D) and 3D CT scan (E&F). Note the coronoid fracture and extension of condyle fracture to the radiocapitellar joint.

The patient was a 19-year-old boy referred to our center with pain and deformity in right elbow after falling from a height of 2 m. Early radiographs showed a fracture of the lateral condyle of the humerus with posterolateral dislocation of the elbow (Figure 1). The patient was treated by closed reduction and long arm splint at another center with the diagnosis of posterolateral elbow dislocation. The patient referred to our center for further treatment. The patient gave us written consent for this report.

The elbow CT scan showed a fracture of the lateral condyle of the elbow as well as a coronoid fracture (type I of Regan Morrey) (Figure 1). The patient was admitted for surgery due to the fracture and instability of the elbow.

**Figure 2.**
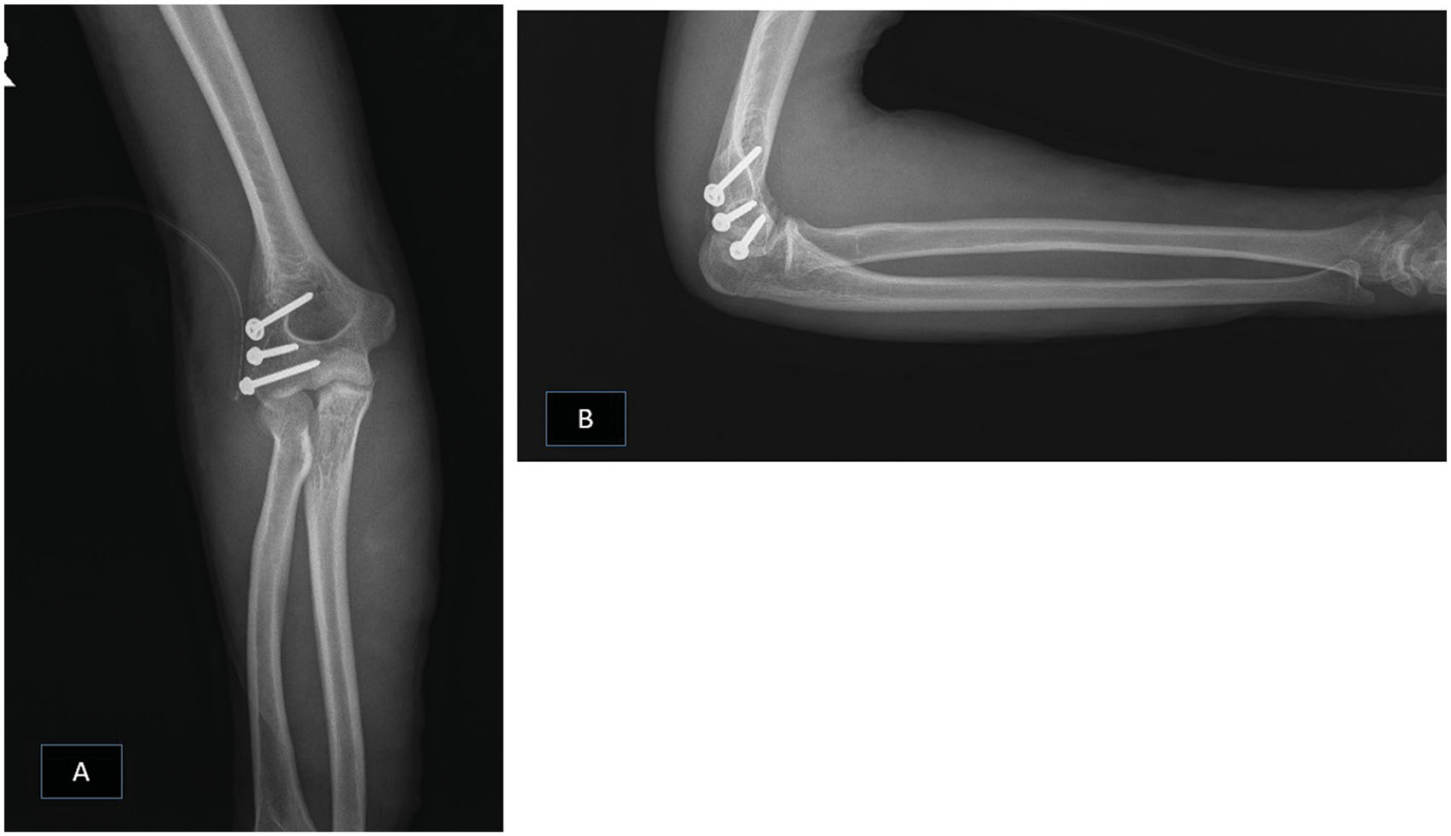
Post-operative AP (A) and lateral (B) radiographs of the patient.

By a direct lateral incision, the brachialis and brachioradialis muscles are shaved about 3 cm above the lateral condyle from the lateral surface of the humerus. Lateral condyle fracture was reduced and fixed with three full-threaded cortical screws (Figure 2). Considering that the lateral collateral ligament (LCL) was intact and attached to the condylar fragment, we hoped that the joint became stable by fixing the fractured fragment. Then, the surgeon examined the elbow by gravity extension test, but the elbow was unstable in extension more than 50° of flexion. Therefore, the incision extended distally by splitting the extensor digitorum communis (EDC) [2], and the anterior capsule with coronoid was exposed. As the coronoid fracture size was small (type I Regan Morrey), the anterior capsule of the elbow was reattached to the ulna with trans-osseous Ethibond No. 5 thread; hence, the elbow became stable after that.

## Discussion

Elbow dislocations are divided into two general categories, simple and complex [3]. Simple dislocations only have capsuloligamentous damage without fractures, while there are concomitant fractures in complex dislocations.

Elbow fractures are categorized into four groups. Group 1: anterior dislocations (trans-olecranon): in this group, olecranon fractures with coronoid fractures cause anterior elbow dislocations while the radius head remains intact. Group 2: posterior olecranon fracture-dislocation or posterior Monteggia’s injury: a comminuted proximal ulna fracture involves the coronoid. The radial head may be fractured. Group 3: terrible triad: the radial head and coronoid fractures due to posterior elbow dislocation. Group 4: varus posteromedial instability: varus elbow instability in addition to a coronoid fracture. This lesion may be associated with LCL damage or fracture of the olecranon [4].

Few studies have recently reported the association of lateral epicondyle fracture with LCL rupture in their patient reports. In 2008, Aksu et al. identified eight patients with elbow dislocations and coronoid fractures; one patient in this study had a lateral epicondylar fracture. At the radiography of this patient, LCL avulsion fracture is evident, but the fracture has not reached the radiocapitellar joint and is a small fragment [5].

Lee et al. [6] studied CT scans and MRIs of 64 patients with posterolateral elbow dislocations. Fifty-four dislocations had associated fractures with eight elbow epicondyle fractures. This study did not report simultaneous epicondyle and coronoid fractures within these eight patients. Of course, the size of the fractured fragment in our patient was big enough to accept three screws, and the fracture extended to the radiocapitellar joint (condylar fracture).

To the best of our knowledge, this type of fracture (lateral condyle fx, intact LCL, intact radial head, posterolateral elbow dislocation and coronoid fracture (type 1 Regan Morrey) needs reconstruction) is not reported in adults previously.

The pathomechanics of this fracture pattern could resemble the posteromedial rotatory instability (PMRI) described by O’Driscoll [7]. This injury is mainly occurred by pronation, varus and axially directed forces to the elbow. This type of injury is rare; its exact incidence is unclear [4]. Typically partial or complete injury of LCL is common. The joint is unstable in varus. A flake fragment may be avulsed by LCL.

In CT scan, an anteromedial coronoid fracture could be recognized [8]. O’Driscoll described three types of coronoid fracture (rim only (I), rim and tip (II), rim, tip and sublime tubercle (III)). This type could be associated with the elbow dislocation, olecranon fracture or rarely radial head fracture [9]. Of course, only a slight elbow incongruity may be present and the lesion could be missed; this could rapidly lead to elbow arthrosis. Then, any isolated coronoid fracture should be further studied by examination and CT scans to assess this type of fracture. Our patient has a coronoid fracture and lateral condyle fracture with elbow dislocation. The coronoid fracture in this patient is assessable by the Regan Morrey classification, but an intact radial head rules out the terrible triad of the elbow.

During surgery, we found the anterior capsule detached from the anterior ulna. We had to reattach the anterior capsule to obtain stability. Then, this pattern of fracture has some specifications of the terrible triad and some of PMRI. We think that the lateral epicondyle fracture is an avulsion fracture. An axial force helped the avulsion force to detach a large intraarticular fragment. Then, we suggest this pattern of fracture can mimic PMRI and it could potentially lead to a broader injury pattern spectrum for elbow fracture-dislocation.

**Figure 3.**
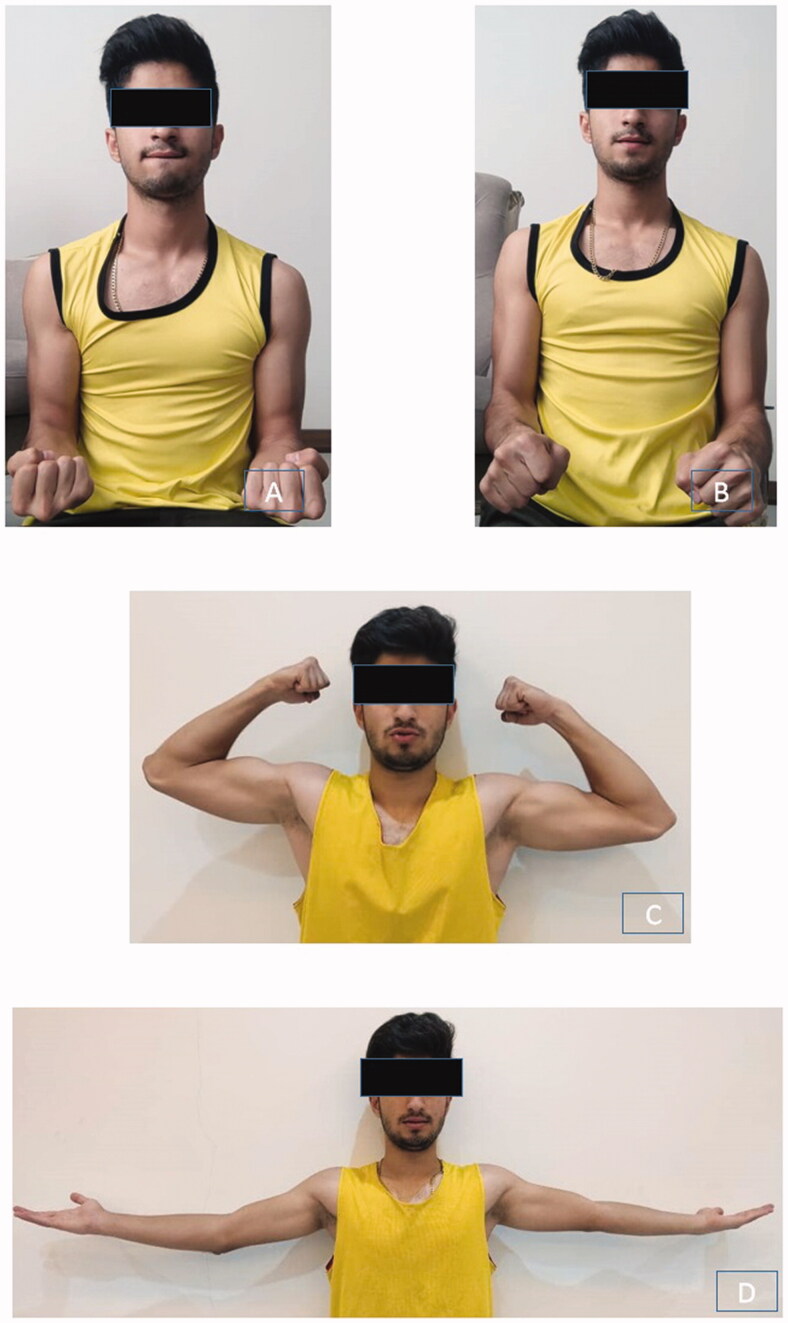
Six months follow-up supination (86°) (A), pronation (86°) (B), flexion (150°) (C) and extension (10°) (D) of the patient.

Eight months after the surgery, the patient has 150° of flexion with 10° of extension and 86° of supination and pronation with no pain (Figure 3).
